# Genotypic Variation under Fe Deficiency Results in Rapid Changes in Protein Expressions and Genes Involved in Fe Metabolism and Antioxidant Mechanisms in Tomato Seedlings (*Solanum lycopersicum* L.)

**DOI:** 10.3390/ijms161226086

**Published:** 2015-11-25

**Authors:** Sowbiya Muneer, Byoung Ryong Jeong

**Affiliations:** 1 Division of Applied Life Science (BK21 Plus), Graduate School, Gyeongsang National University, Jinju 660-701, Korea; sobiyakhan126@gmail.com; 2 Institute of Agriculture & Life Science, Gyeongsang National University, Jinju 660-701, Korea; 3 Research Institute of Life Science, Gyeongsang National University, Jinju 660-701, Korea

**Keywords:** antioxidants, Fe deficiency, Fe metabolism, gene expressions, *Solanum lycopersicum*

## Abstract

To investigate Fe deficiency tolerance in tomato cultivars, quantification of proteins and genes involved in Fe metabolism and antioxidant mechanisms were performed in “Roggusanmaru” and “Super Doterang”. Fe deficiency (Moderate, low and –Fe) significantly decreased the biomass, total, and apoplastic Fe concentration of “Roggusanmaru”, while a slight variation was observed in “Super Doterang” cultivar. The quantity of important photosynthetic pigments such as total chlorophyll and carotenoid contents significantly decreased in “Roggusanmaru” than “Super Doterang” cultivar. The total protein profile in leaves and roots determines that “Super Doterang” exhibited an optimal tolerance to Fe deficiency compared to “Roggusanmaru” cultivar. A reduction in expression of PSI (photosystem I), PSII (photosystem II) super-complexes and related thylakoid protein contents were detected in “Roggusanmaru” than “Super Doterang” cultivar. Moreover, the relative gene expression of *SlPSI* and *SlPSII* were well maintained in “Super Doterang” than “Roggusanmaru” cultivar. The relative expression of genes involved in Fe-transport (*SlIRT1* and *SlIRT2*) and Fe(III) chelates reductase oxidase (*SlFRO1*) were relatively reduced in “Roggusanmaru”, while increased in “Super Doterang” cultivar under Fe deficient conditions. The H^+^-ATPase relative gene expression (*SlAHA1*) in roots were maintained in “Super Doterang” compared to “Roggusanmaru”. Furthermore, the gene expressions involved in antioxidant defense mechanisms (*SlSOD*, *SlAPX* and *SlCAT*) in leaves and roots showed that these genes were highly increased in “Super Doterang”, whereas decreased in “Roggusanmaru” cultivar under Fe deficiency. The present study suggested that “Super Doterang” is better tomato cultivar than “Roggusanmaru” for calcareous soils.

## 1. Introduction

Fe is an essential element for appropriate growth and development of plants [1]. Fe is called a redox metal for physiological and metabolic processes such as photosynthesis, respiration, nitrogen metabolism, hormone biosynthesis, reactive oxygen species scavenging, osmoprotection and pathogenic defense [2,3,4], Fe is the most abundant element on earth but has very low solubility in oxygenated solutions [5,6]. Fe insolubility can cause a deficiency in plants that results in significant losses in agricultural productivity [7,8].

To investigate the Fe deficiency problems, binary Fe acquisition mechanisms (Strategy I and Strategy II) are developed in higher plants [9]. The Strategy II is restricted to graminaceous plants while Strategy I is used by all plants except graminaceous plants. The Strategy II system involves the secretion of phytosiderophores (mugineic acids) and other derivatives such as 2’-deoxymugineic acid (DMA), epi-hydroxymugineic acid (epi-HMA) and 3-epihydroxy 2’-deoxymugineic acid (epi-HDMA) via roots [9,10,11,12,13]. Phytosiderophores are subsequently taken via Yellow stripe transporter 1 (YS1) transporters which belong to OPT (oligopeptide transporter) and the transport of Fe(III) chelate via Yellow stripe transporter 1 (YS1) transporter might be a proton coupled transport [10,11]. The phytosiderophores scavenge Fe, resulting in the formation of soluble Fe^3+^ complexes that can be taken up by active transport mechanisms [12,13]. In Strategy I plants, plasma membrane H^+^-ATPase activity increases to extrude the proton gradient for ion uptake [14]. Strategy I plants not only increase the activity of electrochemical gradient for ion uptake but also mobilize the apoplastic and rhizospheric Fe(III) pools, which are poorly soluble at neutral or alkaline pH.

Tomato is widely used as a model plant for the investigation of Strategy I mechanisms [3,15,16]. The ferric reductase oxidase (FRO) gene encodes the ferric chelate reductase enzyme responsible for reducing Fe [17], and the Fe(II) regulated transporter (*IRT1*/*IRT2*) gene encodes a transporter for Fe^2+^, allowing for its intake into root cells [18]. The putative *IRT1*, *IRT2* and *FRO2* genes have been identified in several Strategy I plants and have been implicated in the up-regulation of Fe availability in conditions of deficiency. Chlorotic lesions are recognized as distinct symptoms of Fe deficiency [19], as well as decreased content of photosynthetic pigments [8,20]. Decreases in photosynthetic pigments lead to a reduction in the granal and stromal lamellae of chloroplasts affecting PSI and PSII, thereby having a negative effect on many thylakoid multiprotein complexes [21]. Important reactions during photosynthesis take place in subcompartments of thylakoids which are known as multiprotein complexes (MCPs). The MCPs are diversified into multi-complex proteins including PSI, PSII, ATP synthase complex and cytochrome *b6*/*f* (*cyt b6/f*) complex. Fe deficiency also leads to oxidative stress primarily due to its functional requirement in multiple protein complexes [22] and as a co-factor in antioxidant enzymes [23]. Oxidative damage arising from Fe deficiency occurs due to the unbalanced generation of reactive oxygen species (ROS) [19,23]. ROS production can be observed in the presence of thiobarbituric acid reactive substances (TBARS), which produces hydrogen peroxide (H_2_O_2_) and singlet oxygen (O_2_^−1^). ROS are an unavoidable byproduct of normal aerobic metabolism [24], but imbalances between ROS production and antioxidative processes by enzymatic and non-enzymatic reactions causes oxidative stress. This manifests as photo-oxidative damage to DNA, proteins, lipids, and ultimately cell death. The Fe deficiency and oxidative damage connection is more evident since Fe plays dual roles in plants cells, either as an antioxidant or a pro-oxidant factor. On the other hand, Fe is also a co-factor for antioxidant enzyme activities and can also act as a pro-oxidant for generation of free radical via Fenton reactions. The various antioxidant enzyme activities such as superoxide dismutase (SOD), ascorbate peroxidase (APX), catalase (CAT), glutathione reductase (GR) and non-enzymatic antioxidants such as GSH (reduced glutathione) and GSSG (oxidized glutathione) are, however, present in plant cells to mitigate oxidative damage [24].

**Figure 1:**
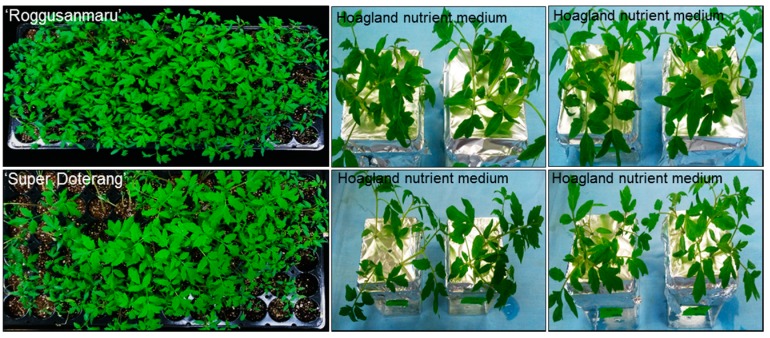
Representative images of tomato cultivars “Roggusanmaru” and “Super Doterang” used in the study.

The aim of this study was to investigate which of the two most used tomato variety candidates (Roggusanmaru and Super Doterang) was better suited for growth under iron limiting conditions (moderate, low, and complete Fe deficiency). These two tomato genotypes are widely used in Korea because of their highest fruit consumption and quality, however, it has not been proven yet which cultivar is suitable to grow under abiotic stresses, especially under Fe deficiency. To achieve this goal, the growth of plants, as well as pigments as biochemical markers of photosynthetic efficiency, and gene expression of some proteins in photosynthesis and markers of iron starvation under controlled conditions with limited iron was evaluated. For our experimental analysis, both cultivars were germinated on square plug tray containing commercial Tosilee medium (Tosilee medium, Shinan Precision Co., Jinju, Korea) and then transferred to Hoagland nutrient medium (Figure 1). Measurements included growth parameters such as fresh and dry biomass, pigment analysis, total protein profile and thylakoid protein expressions. Furthermore, we followed the gene expression analysis of Fe-transport (*SlIRT1* and *SlIRT2*) and Fe(III) chelate reductase oxidase (*SlFRO1*) and genes encoding thylakoid proteins (*SlPSI* and *SlPSII*). We also studied H^+^-ATPase relative gene expression (*SlAHA1*) in the roots. Finally, we investigated the gene expression analysis of antioxidant mechanisms (*SlSOD*, *SlAPX*, and *SlCAT*). From our study, we hypothesize that “Super Doterang” is a more tolerant tomato cultivar than “Roggusanmaru” with refard to Fe deficiency.

## 2. Results

### 2.1. Plant Growth Parameters

**Figure 2:**
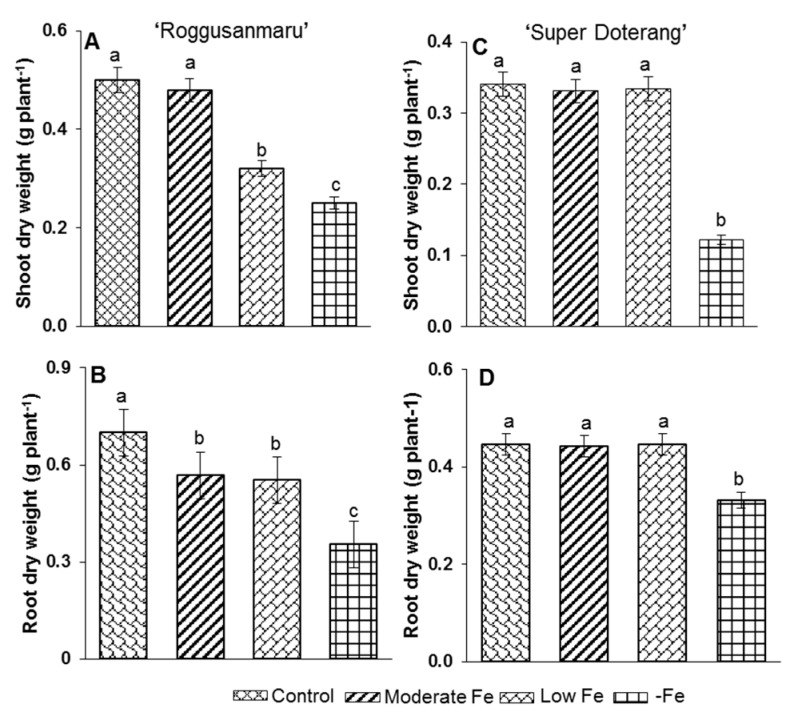
Dry weight in (**A**,**B**) shoot and roots of “Roggusanmaru” and (**C**,**D**) “Super Doterang” tomato cultivar. Plants were grown in Fe-sufficient (control); moderate Fe; low Fe; and Fe-deficient (–Fe) Hoagland nutrient medium for 10 days. Values are Mean ± SE (*n* = 4). Significant differences (*p* ≤ 0.05) among treatments are designated by different letters a, b, c according SAS (statistical analysis software) analysis.

Fe shortage caused no marked effect on the dry weight of “Super Doterang” tomato cultivars, while in “Roggusanmaru” tomato cultivars, dry weight was decreased by 12% in moderate and low Fe deficiency and approximately to 50% under complete Fe deficiency (Figure 2). Plants under complete Fe deficiency showed only a slight decrease in dry weight (−20%) in “Super Doterang”, which was in contrast with the results for “Roggusanmaru”. In “Roggusanmaru”, the dry weight of roots decreased approximately by 50% under Fe deficiency, however, remains constant at moderate and low Fe deficiency.

### 2.2. Total Fe Concentration and Apoplastic Fe Amount

**Figure 3:**
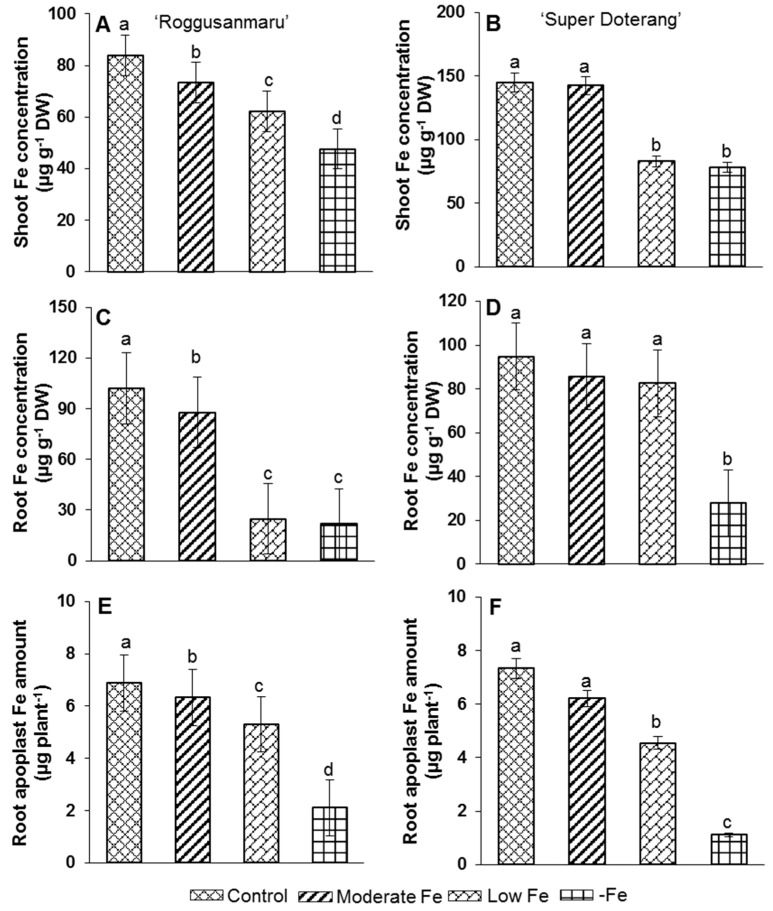
(**A**–**D**) Shoot and root Fe-concentration (**E**,**F**) Fe amount in root apoplast of “Roggusanmaru” and “Super Doterang” tomato cultivars. Plants were grown in Fe-sufficient (control); moderate Fe; low Fe; and Fe-deficient (–Fe) Hoagland nutrient medium for 10 days. Values are Mean ± SE (*n* = 4). Significant differences (*p* ≤ 0.05) among treatments are designated by different letters a, b, c, d according SAS (statistical analysis software) analysis.

Total Fe concentration in “Super Doterang” was shown to be 60% higher than that in “Roggusanmaru” tomato cultivars (Figure 3), especially in the root, under moderate and low Fe treatments. In “Super Doterang”, there was a 12% decrease in Fe concentration under complete Fe deficiency (–Fe) compared with the control. In “Roggusanmaru”, the Fe concentration showed a slight decrease in moderate Fe concentration, a severe reduction in low Fe concentration and the lowest level in complete Fe deficiency (–Fe).

Similarly, root apoplast Fe amount in “Super Doterang” was decreased by 70% and 80% in “Roggusanmaru” (Figure 3), respectively, under complete Fe deficiency compared to Fe sufficient plants. A slight variation was observed among moderate, low, and Fe sufficient “Super Doterang” compared to “Roggusanmaru” tomato cultivars.

### 2.3. Pigment Analysis

**Figure 4:**
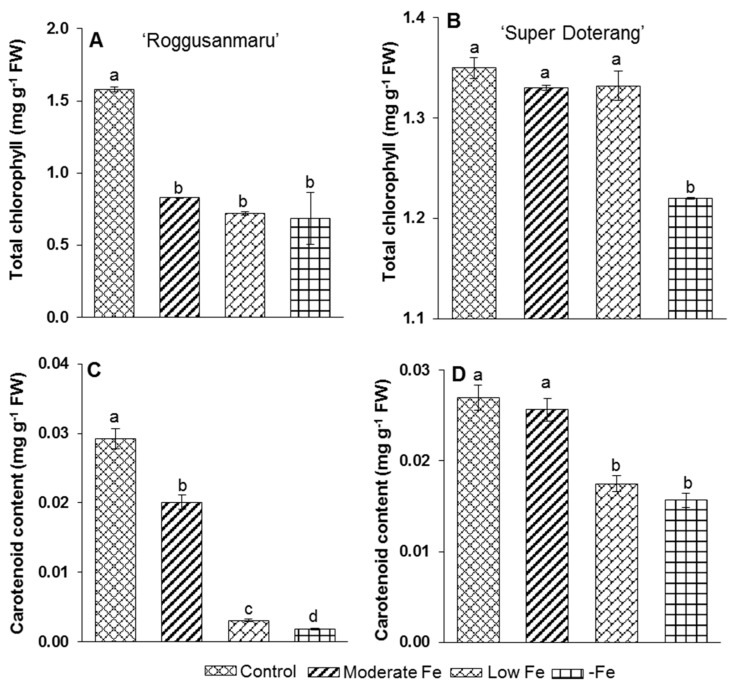
(**A**,**B**) total chlorophyll and (**C**,**D**) carotenoid content in shoots of “Roggusanmaru” and “Super Doterang” tomato cultivar. Plants were grown in Fe-sufficient (control); moderate Fe; low Fe; and Fe-deficient (–Fe) Hoagland nutrient medium for 10 days. Values are Mean ± SE (*n* = 4). Significant differences (*p* ≤ 0.05) among treatments are designated by different letters a, b, c, d according SAS (statistical analysis software) analysis.

Photosynthetic pigments decreased significantly in “Roggusanmaru” compared with “Super Doterang” cultivars (Figure 4). Total chlorophyll decreased by 46% under moderate Fe concentration, 58% under low Fe concentration and showed the highest decrease of 70% under complete Fe deficiency (–Fe) with “Roggusanmaru” (Figure 4A). In contrast, with “Super Doterang”, total chlorophyll content decreased only under complete Fe deficiency (–Fe) and remained the same in the moderate and low Fe deficiency treatment conditions (Figure 4B).

There was a similar pattern for carotenoid content. In “Roggusanmaru” (Figure 4C), carotenoid content decreased 30% under moderate Fe deficiency, 40% under low Fe deficiency and 93% under complete Fe deficiency (–Fe). In “Super Doterang” (Figure 4D), there was only a slight variation in carotenoid content at low and moderate Fe concentrations and a 37% reduction under complete Fe deficiency (–Fe).

### 2.4. Total Protein Profile Content

**Figure 5:**
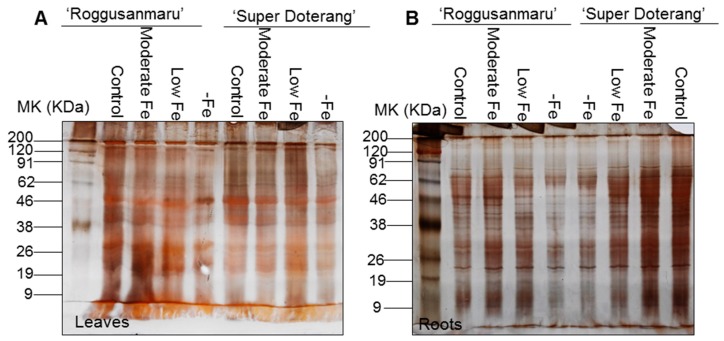
Total protein profile in (**A**) leaves and (**B**) roots of “Roggusanmaru” and “Super Doterang” tomato cultivar. Plants were grown in Fe-sufficient (control); moderate Fe; low Fe; and Fe-deficient (–Fe) hydroponic media for 10 days. Proteins extracted from shoots and roots were run on 12.5% polyacrylamide gel electrophoresis and stained with silver stain (MK on gels denote molecular marker).

The total protein profile in the leaves of both tomato cultivars (Figure 5A) showed that for “Roggusanmaru” the intensities of several protein bands such as RuBisCO (Ribulose-1,5-bisphosphate carboxylase/oxygenase) at 50 KDa and proteins at lower molecular weights (9–26 KDa) was decreased under low and –Fe treatment conditions compared with the control, while for “Super Doterang” it was equal to the control conditions except for complete Fe deficiency (–Fe). 

A similar pattern was observed for the total protein profile of the roots in both tomato cultivars (Figure 5B). For “Roggusanmaru”, the intensities of different protein bands such as bands between 9 and 19 KDa and 38–62 KDa was decreased severely under moderate, low and –Fe treatment conditions compared with the control (Figure 5B). For “Super Doterang”, the moderate and low Fe treatment protein band intensity did not show any changes compared with the control and the only variation was with complete Fe deficiency (–Fe).

### 2.5. Changes in Multiprotein Complex Proteins (MCPs) (1D-BN-PAGE)

**Figure 6:**
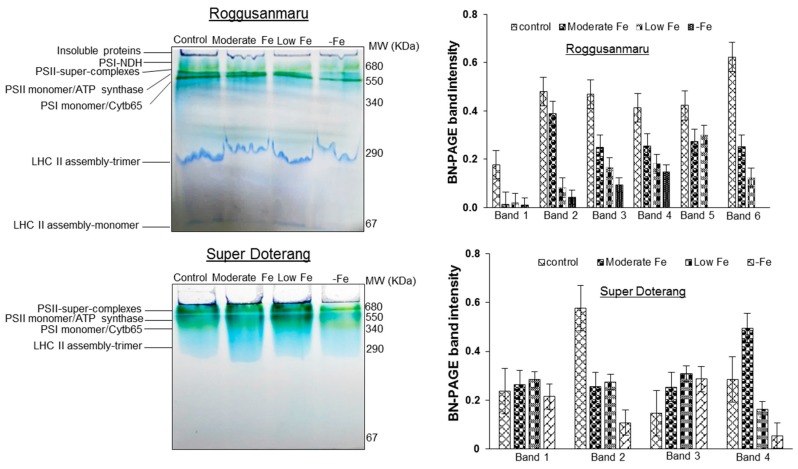
Multiprotein thylakoid membrane proteins (BN-PAGE) expressions and their band intensity in shoots of “Roggusanmaru” and “Super Doterang” tomato cultivar. Plants were grown in Fe-sufficient (control); moderate Fe; low Fe; and Fe-deficient (–Fe) Hoagland nutrient medium for 10 days. Proteins extracted from leaves were run on 7%–12% gradient polyacrylamide gel electrophoresis for blue native page (BN-PAGE). In bar diagram of “Roggusanmaru” Band 1 indicates Psi-NDH, Band 2 indicates PSII-super complexes, Band 3 indicates PSII monomer/ATP synthase, Band 4 indicates PSI monomer/Cytb65, Band 5 indicates LHC (light harvesting complex) II assembly-trimer and Band 6 indicates LHC II (light harvesting complex) assembly monomer. While in bar diagram of “Super Doterang” Band 1 indicates PSII-super complexes, Band 2 indicates PSII monomer/ATP synthase, Band 3 indicates PSI monomer/Cytb65 and Band 4 indicates LHC II (light harvesting complex) assembly-trimer (MW in figures denote molecular weight).

First-dimensional electrophoresis run under native conditions on BN-PAGE with three biological replicates was used to isolate thylakoids (multiprotein complexes) from tomato leaves. The protein profile of the thylakoid MCPs extracted from leaves under different treatments is shown in Figure 6A (control, moderate Fe, low Fe, and –Fe conditions).

The comparative analysis of MCPs among different treatments in each protein band was analyzed semi-quantitatively (shown in bar diagrams). The protein band between 1000–680 KDa comprises of three super complexes of PSI-PSII, and these were in lower quantities in “Roggusanmaru” compared with “Super Doterang”. The intensity of the dark green band at 340 KDa, which was observed as PSII-monomer/ATP synthase, was faint in “Roggusanmaru” under all Fe treatment conditions, while the lowest expression was observed only under –Fe deficiency conditions in “Super Doterang”. The light green band at 290 KDa comprises PSI-monomer/cytochrome b_6_f (band 3). The expression level of PSI-monomer/cytochrome b_6_f protein was more marked in “Roggusanmaru” compared to “Super Doterang” under –Fe treatments. The LHC trimers (light harvesting complex) (band 4) at 140 KDa and dark blue band (LHC monomers, band 5) at 67 KDa were almost the same in all treatment sets compared with the controls.

### 2.6. Effect of Fe-Deficiency on SlPSI and SlPSII

**Figure 7:**
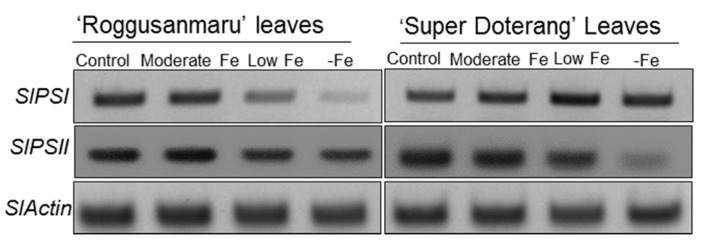
Semi-quantitative RT-PCR analysis of *SlPSI* and *SlPSII* in leaves of “Roggusanmaru” and “Super Doterang” tomato cultivar (*SlActin* acted as a normalized housekeeping gene). Plants were grown under Fe-sufficient (control); moderate Fe; low Fe; and Fe-deficient (–Fe) Hoagland nutrient medium for 10 days.

The multiprotein complex proteins include two important Fe-containing proteins, PSI and PSII. In addition to the expression of PSI and PSII shown in BN-PAGE, further confirmation of their expression was provided by semi-quantitative real-time polymerase chain reaction (RT-PCR) (Figure 7). The relative expression of *SlPSI* and *SlPSII* in “Roggusanmaru” showed reductions under low and –Fe treatments compared with the control conditions. The relative expression of in “Super Doterang” did not show any changes compared with the control under the three Fe treatment conditions (moderate, low and –Fe) except a reduction for *SlPSII* in –Fe treatment conditions.

### 2.7. Effect of Fe-Deficiency on Fe-Transport and Fe(III)-Chelate Activity

**Figure 8:**
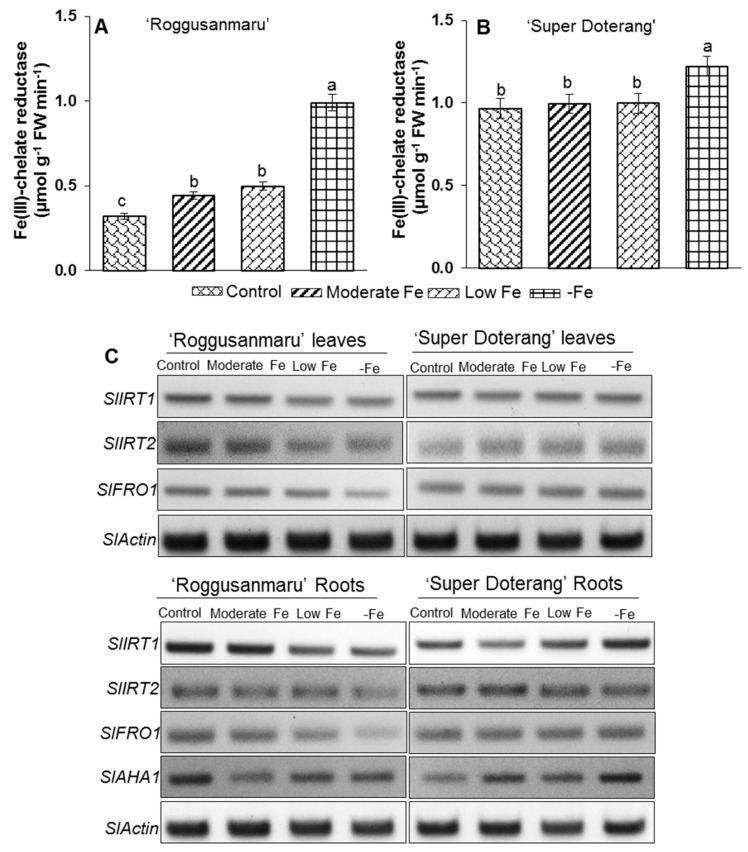
(**A**,**B**) Fe(III) chelate reductase activity in roots and (**C**) semi-quantitative RT-PCR analysis of Fe transport *SlIRT1*, *SlIRT2* and Fe(III) chelate reductase *SlFRO1*, *SlAH1* related genes in leaves and roots of “Roggusanmaru” and “Super Doterang” tomato cultivar (*SlActin* acted as a normalized housekeeping gene). Plants were grown in Fe-sufficient (control); moderate Fe; low Fe; and Fe-deficient (–Fe) Hoagland nutrient medium for 10 days. Significant differences (*p* ≤ 0.05) among treatments are designated by different letters a, b, c according SAS analysis.

The Strategy I plants respond to Fe-shortage by increasing the activity of Fe(III) chelate reductase activity, H+-ATPase and Fe regulated transporters (IRT). Thus, we measured Fe(III) chelate reductase activity in the roots of the tomato cultivars. The activity of Fe(III) chelate reductase activity in “Roggusanmaru” remained the highest in –Fe and the lowest in Fe-sufficient conditions (Figure 8A). The activity of Fe(III) chelate reductase oxidase in “Super Doterang” was significantly higher in –Fe conditions (Figure 8B), whereas no significant difference was observed among the control, moderate, and low Fe conditions.

The expression level of Strategy I genes (*SlIRT1* and *SlIRT2*), was studied in both cultivars grown under moderate, low, and –Fe conditions. The expression level of *SlIRT1* remained unchanged in the leaves of both cultivars, while it lowered in the roots of “Roggusanmaru” under low and –Fe treatment conditions but gradually increases in “Super Doterang” roots (Figure 8C). On the contrary the relative expression of *SlIRT2* decreased under low and –Fe treatment of Roggusanmaru’ and gradually increases under Fe deficiency in “Super Doterang” leaves and roots (Figure 8C).

The response of a gene involved in Fe reduction (*SlFRO1*) and a H^+^-ATPase related gene (*SlAH1*) showed the lowest expression in –Fe conditions in “Roggusanmaru” compared with the control (Figure 8C) and adequate expression was observed in plants grown under moderate levels of Fe. In contrast, the relative expression of *SlFRO1* and *leAHA1* in “Super Doterang” increased slightly under moderate and low Fe conditions and the highest expression was observed under –Fe conditions.

### 2.8. Effect of Fe-Deficiency on Antioxidant Mechanisms

**Figure 9:**
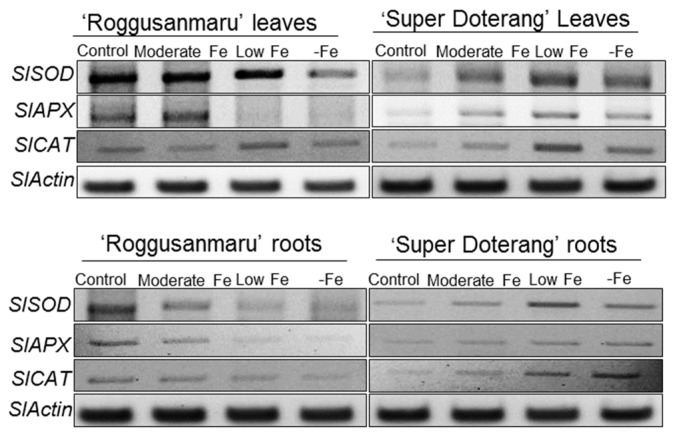
Semi-quantitative RT-PCR analysis of antioxidant mechanism related genes (*SlAPX*, *SlSOD* and *SlCAT*) in shoots and roots of “Roggusanmaru” and “Super Doterang” tomato cultivar (*SlActin* acted as a normalized housekeeping gene). Plants were grown in Fe-sufficient (control); moderate Fe; low Fe; and Fe-deficient (–Fe) Hoagland nutrient medium for 10 days.

The thiobarbituric acid reactive substances (TBARS) provided the evidence that ROS was induced in both cultivars of tomato (Figure S1, Supplementary Material). The key genes involved in antioxidant mechanisms for detoxification of ROS, *SlSOD*, *SlAPX* and *SlCAT*, were studied in the roots and leaves of both cultivars. The expression level of *SlAPX* in the leaves and roots of “Roggusanmaru” was observed only in the control and moderate Fe conditions, while no expression was observed in low and –Fe conditions (Figure 9). On the contrary, the expression level of *SlAPX* in “Super Doterang” was observed highest in low and –Fe leaves and roots.

The expression level of *SlSOD* in the leaves and roots of “Roggusanmaru” decreased, whereas increased in “Super Doterang” under low and –Fe conditions. A similar result was observed for *SlCAT* except for “Roggusanmaru” leaves where expression level was increased in low and –Fe conditions. The expression level of *SlCAT* in roots of “Roggusanmaru” decreased, whereas increased in “Super Doterang” under low and –Fe conditions.

## 3. Discussion

The tomato plant is a suitable model plant for the study of Fe-related nutritional strategies because it belongs to the Strategy I category [3,15,16]. In parallel, several physiological responses of Fe deficiency such as photosynthetic pigment loss (PSI and PSII) [25] in Strategy I and Strategy II plants have been studied [26,27,28,29]. The objective of this study was to investigate the effects of Fe deficiency (moderate, low, and –Fe) on total protein profile, and gene expression analysis involved in Fe metabolism and antioxidant mechanisms in tomato cultivars (Roggusanmaru and Super Doterang). We first analyzed the biomass of plants in our experiments under three Fe treatment regimens and observed that shoot and root biomass decreased in “Roggusanmaru” cultivars (Figure 2) compared to “Super Doterang”. The decrease of biomass in “Roggusanmaru” indicated the sensitivity of plants towards Fe deficiency compared to “Super Doterang” cultivars. The reduction in biomass in “Roggusanmaru” also suggest that interactions between Fe deficiency and shoots/roots lead to physiological alterations which in turn affect water balance, an observation previously reported in a number of studies [6,12], whereas a slight biomass reduction in “Super Doterang” exhibited tolerance to Fe deficiency. We observed that photosynthetic pigments (total chlorophyll and carotenoid content) decreased in Fe-deficient “Roggusanmaru” (Figure 4), whereas this effect was abrogated in “Super Doterang”, underlining a superior ability to tolerate Fe deficiency. We also observed that the total and apoplastic Fe decreased more in “Roggusanmaru” compared with “Super Doterang” (Figure 3). The reduction of Fe concentration was, however, recovered by the induction of ferric chelate reductase oxidase besides the expression of Fe^2+^ transporter *IRT1*. Our observations show that “Super Doterang” has a significant ability to maintain its nutritional homeostasis by maintaining the concentration of total and apoplastic Fe under mineral deficient conditions, especially for Fe, compared with “Roggusanmaru”.

We also observed that Fe deficiency reduced overall protein profile to a greater degree in the leaves and roots of “Roggusanmaru” than in those of “Super Doterang” (Figure 5). This reduction in several protein band intensities in “Roggusanmaru” might be due to a reformist inactivation of biochemical pathways concomitant with signal transduction and gene regulation, eventually stalling protein synthesis mechanisms [19,30] and it could be associated with excessive ROS production affecting the proper folding or assembly of proteins, leading to protein degradation [4,31]. Given that the chloroplast is an organelle that is highly susceptible to abiotic stress, we carried out parallel work using BN-PAGE for integral thylakoid proteins and observed significant reductions in PSI (RCI + LHCI), PSII, cytochrome b_6_f and LHC-II trimer complex (Figure 6A) under Fe-deficient conditions in “Roggusanmaru” and that this loss was largely absent in “Super Doterang”. These results were confirmed by semi-quantitative RT-PCR analysis of two important chloroplast proteins, *SlPSI* and *SlPSII* (Figure 7). The decrease in chloroplast proteins suggests that “Roggusanmaru” is more sensitive cultivar than “Super Doterang” to Fe deficiency. Analogous observations were made in Spinach [20] and in lower organisms [32]. The decrease in chloroplast proteins might be also due to decrease in chlorophyll molecules and due to impaired chloroplast structure [33].

To address the question of whether conditions of Fe deficiency in “Super Doterang” activate mechanisms for improving Fe uptake, the plasma membrane-H^+^-ATPase relative expression (*leAHA1*), the expression of Fe^2+^ transporter (*SlIRT1* and *SlIRT2*) and Fe(III) chelates reductase oxidase (*SlFRO1*) genes in the roots and leaves were analyzed (Figure 8). Fe deprivation elicited no change in the expression level of *SlIRT1* in the leaves of both cultivars. Although *SlIRT1* lowered in the roots of “Roggusanmaru” but gradually increased in “Super Doterang” roots. This might be due to the reason that Fe deficiency endured the steady position to some extent in the leaves compared to the roots by not changing the expression level of *SlIRT1*. On the other hand the relative expression of *SlIRT2* decreased under low and –Fe treatment of Roggusanmaru’ and gradually increases under Fe deficiency in “Super Doterang” leaves and roots (Figure 8C). The lower expression of relative genes might be due to cell damage in leaf tissue from the reduction of photosynthetic proteins (Figure 6A). This knocks-out certain genes and in turn also results in a reduction of photosynthetic activities such as stomatal conductance and transpiration [34]. It can thus be suggested that “Super Doterang” is a more tolerant genotype than “Roggusanmaru” to Fe-deficiency.

**Figure 10:**
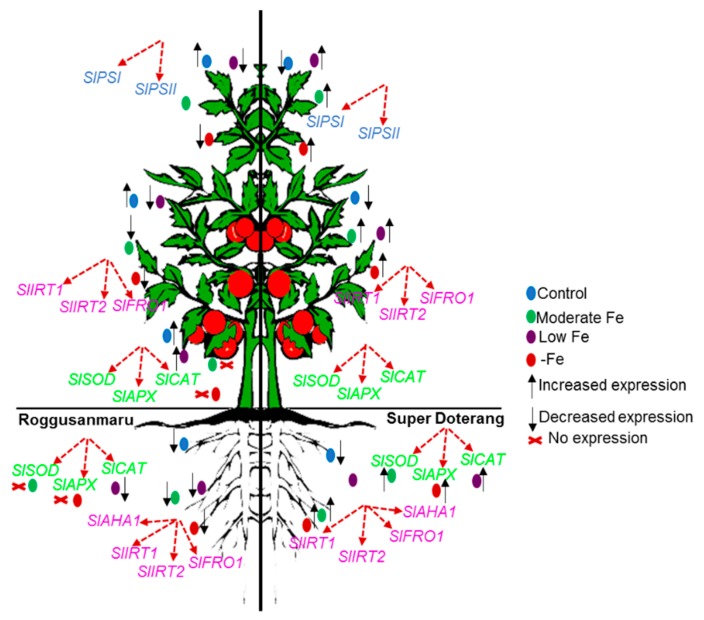
Diagrammatic representation of the present study for the regulation and uptake of Fe in selected tomato cultivars (Roggusanmaru and Super Doterang). Expression of Fe transport and Fe(III) chelate related genes, as well as antioxidant mechanism is represented. Upper arrows represent increased fold change, lower arrows represent lower fold change and cross represents no fold changes.

Oxidative damage is a major cytotoxic consequence of reactive oxygen species (ROS) generation. Our results suggest that Fe deficiency results in oxidative damage in both cultivars of tomato, indicated by TBARS, a phytotoxic marker of reactive oxygen species (ROS) damage [35,36] (Figure S1, Supplementary Material). The SOD enzyme plays an important role in the ascorbate-glutathione cycle by mediating the dismutation of superoxide anions to produce H_2_O_2_ [37]. Our results show that the relative expression of *SlSOD* under Fe deprivation is decreased in “Roggusanmaru”, which suggests a failure in ability to quench ROS production. This might be due to a low availability of cysteine or the failure or limited availability of Fe-chelates [8] (Figure 9). Catalase is another important antioxidant and, as a heme-containing compound, is likely to be more affected by Fe deficiency [38,39]. Fe deprivation caused a decrease in the relative expression of *SlCAT* in “Roggusanmaru” while increasing its expression in “Super Doterang”. Similarly, the antioxidant enzyme APX plays an important role in ROS control, and associates with glucose through the pentose phosphate pathway and NADPH to generate the reduced form of GSH from the oxidized disulfide form (GSSG). We observed that the relative expression of *SlAPX* was severely decreased in “Roggusanmaru”. This may be a result of the high demand for Fe from the APX molecule, as it has a non-heme Fe atom in addition to a heme group [40]; however, the relative expression of *SlAPX* was found to be increased in “Super Doterang”, which might be attributable to limitations in heme-catalyzed damage to the APX protein. Our results suggested that “Super Doterang” a promising cultivar to Fe-deficiency tolerance (for overall conclusions of the results please see Figure 10). It was also shown that “Roggusanmaru” is a susceptible cultivar that needs to be adapted to growing conditions other than calcareous soils.

## 4. Material and Methods

### 4.1. Plant Materials and Growth Conditions

A screening with five different cultivars of tomato (*Solanum lycopersicum* L.) viz. Roggusanmaru, Super Doterang, Mini Tomato, Osec Tomato, and Goldsan Cherry was performed to select the final two cultivars for this study. The major parameter considered was seed germination for cultivar selection since germination rate is important for improved crop production. Besides, the crop growers (farmers) also select seeds based on germination rate for better crop production. As “Roggusanmaru” and “Super Doterang” were the cultivars with higher germination rate, thus were selected for the present study. After germination, seedlings with uniform size were transferred to Hoagland nutrient medium, followed by dividing the plants into four groups receiving different treatments of Fe: sufficient in Fe (20 µM Fe-(II) EDTA “Ethylenediaminetetraacetic acid”) (control), moderate sufficiency in Fe (10 µM Fe-(II) EDTA) (moderate Fe), low Fe sufficiency (5 µM Fe-(II) EDTA) (low Fe) and Fe-deprived (–Fe) with four biological replicates. Plants were grown in a 300 mL magenta box (four plants in one magenta box represent one replicate) with a hydroponic nutrient solution containing (mM for the macro elements): 1.0 NH_4_NO_3_; 0.4 KH_2_PO_4_; 3.0 CaCl_2_; 1.5 MgSO_4_; 0.15 K_2_HPO_4_; and Fe(III)EDTA (20 µM for Fe-sufficient plants; while 10 and 5 µM for moderate and low Fe concentration, respectively) and (µM for the micro elements): 14 H_3_BO_3_; 5.0 MnSO_4_∙H_2_O, 3.0 ZnSO_4_∙7H_2_O; 0.7 CuSO_4_∙5H_2_O; 0.7 (NH_4_)6MO_7_O_2_; and 0.1 COCl_2_. The nutrient solution was continuously aerated and renewed every 3 days and pH of the nutrient media was continuously maintained to 5.8 with 1.6 µS electrical conductivity throughout the growth period. For complete Fe-deficiency, the Fe was completely removed from a hydroponic solution by avoiding the addition of Fe(III)-EDTA. The white fluorescent light was supplemented (100 µmol·m^−2^∙s^−1^) at the canopy height for 16 h∙day^−1^. The relative temperature was set to 25 °C. The leaves and roots of plants were carefully excised after 10 days of treatment and frozen in liquid nitrogen followed by storage in deep freezer (−80 °C). The plants were also dried for 48 h for chemical analysis.

### 4.2. Biomass Analysis

For fresh biomass analysis plants were blotted dry on a lint-free tissue paper and each part of the plant were carefully separated and weighed on weighing balance. The plant materials were also dried in an oven for 48 h at 70 °C for dry mass analysis.

### 4.3. Analysis of Fe Concentration and Root Apoplastic Fe-Amount

Fe concentrations in experimental plants were determined by inductively coupled plasma optical emission spectrometry, (ICP-OES, Thermo Elemental—Iris Advantage, Waltham, MA, USA) as described in our previous studies [8]. The apoplastic Fe content was analyzed spectrophotometrically according to the method of Bienfait *et al.* [41].

### 4.4. Pigment Determination

Total chlorophyll and carotenoid content were determined by dimethyl sulfoxide (DMSO) as earlier described by Hiscox and Israclstam [42] and calculations of pigments were obtained using the formulae given by Arnon [43].

### 4.5. Total Protein Profile by SDS-PAGE (Sodium Dodecyl Sulfate Polyacryalamide Gel Elctrophoresis)

The frozen plant samples (leaf and root) were grinded in liquid nitrogen with pestle and mortar to a fine powder. The powdered samples were used for extraction of protein using protein extraction buffer containing 40 mM (*w*/*v*) Tris-HCl, pH 7.5, 2 mM (*w*/*v*) EDTA, 0.07% (*w*/*v*) β-mercaptoethanol, 2% (*w*/*v*) PVP (polyvinylpyrrolidone) and 1% (*v*/*v*) Triton X-100. The extracts was centrifuged at 13,000 rpm for 10 min at 4 °C and resulting supernatant was mixed with 2-X protein-dye containing 240 mM Tris-HCl (pH 6.8), 40% glycerol, 8% SDS, 0.04% bromophenol blue and 5% beta-mercaptoethanol. The samples containing 10 μg of proteins quantified by Bradford [44] using BSA (bovine serum albumin) as a standard curve were loaded on 12.5% polyacrylamide gel (Bio-Rad, Hercules, CA, USA). Subsequently, the gels were stained in commercial accessible silver stain according to manufacturer’s instructions (Bio-Rad, Hercules, CA, USA).

### 4.6. Analysis of Multiprotein Complex Proteins in Thylakoids (MCPs) (First-Dimensional Blue Native Page) (1D-BN-PAGE)

1D BN-PAGE thylakoid proteins was evaluated according to our previous studies [45]. The electrophoresis was performed at 4 °C in a Protean II xi cell electrophoresis system (Bio-Rad) for first dimension at a constant voltage of 100 V for 5–6 h and then steadily increasing up to 200 V till the run was complete. The molecular weight of proteins was calculated according to previous studies [46].

### 4.7. Image Analysis

First dimension gel images were photographed using a high-resolution digital camera (Canon G10, Tokyo, Japan) at a default resolution of 300 dpi. Image analysis was carried out with GelQuantNET software (BiochemLabSolutions.com, San Francisco, CA, USA) for protein band quantification. 

### 4.8. Determination of Fe^3+^-EDTA Reduction Activity

The Fe(III) chelate reductase activity was determined as previously given by Schmidt [47]. Three independent measurements in the presence or absence of Fe(III)-EDTA were performed, values were calculated as µmol Fe^2+^ (g^−1^∙FW∙min^−1^).

### 4.9. Isolation of RNA, cDNA Preparation and Semi-Quantitative RT-PCR

The RNA isolation was performed in leaves and roots using RNA isolation kit according to the manufacturer's instructions (Intron Biotechnology, Seongnam-City, Korea). The isolated RNA from leaves and roots were quantified by using nano-drop spectrophotometer and 1 µg of DNAase-treated RNA was used to synthesize cDNA using a reverse transcriptase kit (Promega, Madison, WI, USA). Semi-quantitative RT-PCR was performed using a Gene Amp* PCR system 9700 (Applied Biosystems, Foster City, CA, USA) for 5 min at 95 °C, followed by 25 cycles consisting of 20 s at 95 °C, 30 s at 58 °C and 30 s at 72 °C, then 10 min at 72 °C. All quantifications were normalized to actin expression. The amplified products were run on 1% agarose gel and were stained with ethidium bromide (Sigma-Aldrich, St. Louis, MO, USA). Several gene-specific primers were designed for the semi-quantitative RT-PCR and the primers with a highest specificity were selected for gene amplification. The gene specific primers used in this study are listed in Table 1.

**Table 1:** List of primers used to quantify gene expression levels.

Gene	Accession Number	Forward Primer	Reverse Primer
*SlPSI*	DQ525206.1	5′-ATAAGGGTCTTTATGACACA-3′	5′-TATAATGCAGCTTGAGTAGT-3′
*SlPSII*	NM_001247113.2	5′-ATGGCAAGCACAGTAATGAG-3′	5′-TCTCAAGGCCATGCTTCCAT-3′
*SlIRT1*	AF136579.1	5′-TATTTTGTAAAATCCAGATA-3′	5′-ACAATATTTTTTGAATAGTG-3′
*SlIRT2*	AF136580.1	5′-ATTCCTACTCAAAATTCAAA-3′	5′-ACAAACTCCGATCATACTAG-3′
*SlFRO1*	NM_001247400.1	5′-ATGGCTCAAACATCCTCTTC-3′	5′-GAGAGGAAATGTGTTCATCA-3′
*SlAHA1*	M60166.1	5′-AAATCTTGTCCTCTTTTCTT-3′	5′-AAAATCCTGCCAGTCCGGTG-3′
*SlAPX*	NM001247702	5′-AAGGCCACATTCTGTCATCC-3′	5′-TCAAATTTCAGCCACTGCAC-3′
*SlSOD*	M37151	5′-CTGGACTTCACGGGTTTCAT-3′	5′-CCCGGAGAGGAGGGTAAATA-3′
*SlCAT*	NM001247898	5′-TGCATTGAAACCAAATCCAA-3′	5′-CAACACCAATCGACCAACTG-3′
*SlActin*	FJ532351.1	5′-ATGACTCAAATCATGTTTGAG-3′	5′-GCAGCATGAAGATTAAGGTA-3′

### 4.10. Statistical Analysis

The experiments were done using four biological replicates. The Tukey’s studentized range test was employed using SAS software. The results are presented as the Mean ± standard error (SE) with a significance level set at *p* < 0.05.

## Supplementary Materials

Supplementary materials can be found at http://www.mdpi.com/1422-0067/16/12/26086/s1.

## Abbreviations

BN-PAGE (blue native page); IRT (Fe regulator transport); FRO (ferric chelates reductase oxidase); SOD (superoxide dismutase); APX (ascorbate peroxidase); CAT (catalase); ROS (reactive oxygen species); TBARS (thiobarbituric acid reactive substances); SDS-PAGE (sodium dodecyl sulfate polyacrylamide gel electrophoresis); MCP (multiprotein complex proteins); RT-PCR (real time polymerase chain reaction).
