# Supplementary material for: Genotypic Variation under Fe Deficiency Results in Rapid Changes in Protein Expressions and Genes Involved in Fe Metabolism and Antioxidant Mechanisms in Tomato Seedlings (Solanum lycopersicum L.)
Source: Int J Mol Sci. 2015 Nov 25;16(12):28022–37. doi: 10.3390/ijms161226086 (PMC4691033; doi:10.3390/ijms161226086)
Supplement: Supplementary file 1 [file ijms-16-26086-s001.pdf]

Supplementary Materials: Genotypic Variation under Fe Deficiency Results in Rapid Changes in Protein Expressions and Genes Involved in Fe Metabolism and Antioxidant Mechanisms in Tomato Seedlings (*Solanum lycopersicum* L.)

Sowbiya Muneer and Byoung Ryong Jeong

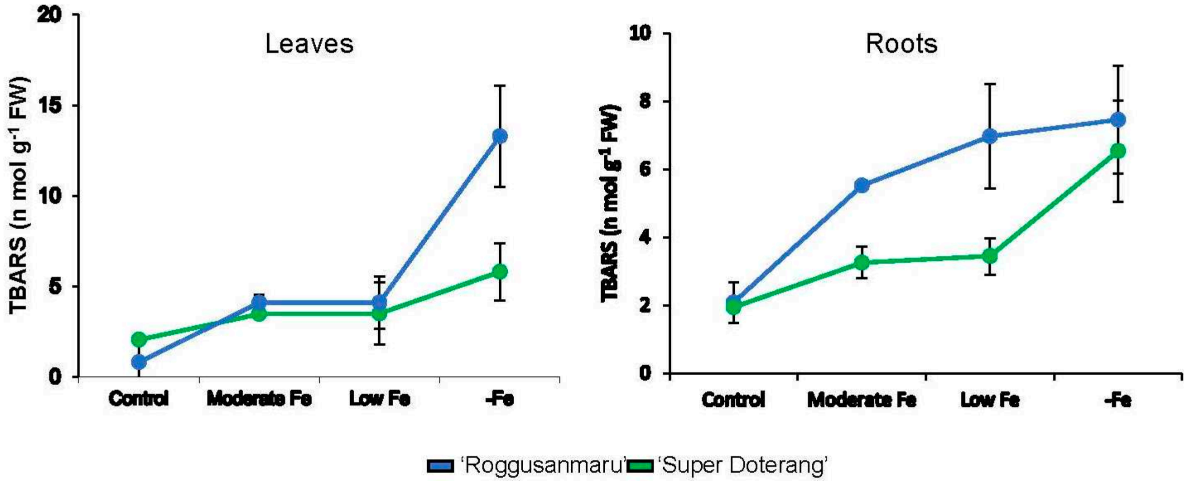

Figure S1. Oxidative damage in the form of thiobarbituric acid reactive substances (TBARS) in "Roggusanmaru" and "SuperDoterang" tomato cultivars. Plants were grown in Fe-sufficient (control); moderate Fe; low Fe; and Fe-deficient (–Fe) hydroponic conditions for 10 days. Color lines represent Mean ± SE of four biological replicates.
